# Extension of the classical classification of β-turns

**DOI:** 10.1038/srep33191

**Published:** 2016-09-15

**Authors:** Alexandre G. de Brevern

**Affiliations:** 1 INSERM, U 1134, DSIMB, F-75739 Paris, France; 2 Univ Paris Diderot, Sorbonne Paris Cité, UMR_S 1134, F-75739 Paris, France; 3 Institut National de la Transfusion Sanguine (INTS), F-75739 Paris, France; 4 Laboratoire d’Excellence GR-Ex, F-75739 Paris, France

## Abstract

The functional properties of a protein primarily depend on its three-dimensional (3D) structure. These properties have classically been assigned, visualized and analysed on the basis of protein secondary structures. The β-turn is the third most important secondary structure after helices and β-strands. β-turns have been classified according to the values of the dihedral angles φ and ψ of the central residue. Conventionally, eight different types of β-turns have been defined, whereas those that cannot be defined are classified as type IV β-turns. This classification remains the most widely used. Nonetheless, the miscellaneous type IV β-turns represent 1/3^rd^ of β-turn residues. An unsupervised specific clustering approach was designed to search for recurrent new turns in the type IV category. The classical rules of β-turn type assignment were central to the approach. The four most frequently occurring clusters defined the new β-turn types. Unexpectedly, these types, designated IV_1_, IV_2_, IV_3_ and IV_4_, represent half of the type IV β-turns and occur more frequently than many of the previously established types. These types show convincing particularities, in terms of both structures and sequences that allow for the classical β-turn classification to be extended for the first time in 25 years.

The functional properties of a protein primarily depend on its three-dimensional (3D) structure. These properties have classically been assigned, visualized and analysed on the basis of protein secondary structures, which are composed of repetitive parts (α-helices^1^ represent 1/3^rd^ of residues, and β-strands^2^ represent 1/5^th^ of residues) connected by coils^3^. This simplification of 3D structure into a unidimensional representation of secondary structure is often regarded as a resolved question. In fact, this simplification conceals the difficulty of precisely defining and assigning repetitive structures^4^, thus explaining the large number of alternative assignment approaches^5^,^6^,^7^,^8^,^9^,^10^,^11^. For instance, comparison of different approaches emphasizes their major discrepancies^12^,^13^. Another limitation of this type of simplification is that the coil state is neglected, although it represents almost 50% of all residues and a large set of distinct local protein structures. Loop analyses cannot provide a complete representation of the coil state because their classification is usually limited to 8 residues^4^,^14^,^15^,^16^,^17^. More precise descriptions are needed to comprehensively describe their diversity.

Helical and extended regions are the most frequently occurring repetitive structures. However, two other local protein conformations have also been characterized: the polyproline II helix and turns. The former is a left-handed helical structure with an overall shape resembling a triangular prism. It represents 5% of all protein residues^18^, contributes to coiled coil super secondary structure formation and is present in fibrous proteins^19^,^20^. Because polyproline II helices do not have strong hydrogen bond patterns, they have not been studied in as much detail as the other local conformations^21^,^22^,^23^,^24^,^25^,^26^.

Turns comprise *n* consecutive residues (denoted *i* to *i*+*n*), in which the distance between Cα(s) of residues *i* and *i*+*n* must be smaller than 7 Å (or 7.5 Å, according to some authors^27^,^28^). The turns are composed of γ-turns (*n* = 3)^29^,^30^, β-turns (*n* = 4), α-turns (*n* = 5)^31^,^32^ and π-turns (*n* = 6)^33^,^34^. The restrictive distance between Cαs applies a particular geometry to the backbone, thereby causing it to turn back on itself.

β-turns have been the most analysed among the turn conformations. Apart from the distance between Cαs, a second rule applies to the characterization of their secondary structure; because helices can easily be confused with a succession of turns, the central residues of β-turns, *i*.*e*., *i*+1 and *i*+2, should not be helical. Similarly, β-turn residues must not consist solely of β-strand residues. β-turns have been classified according to the values of their central residue dihedral angles, φ and ψ. A deviation of ± 30° from these canonical values is allowed on 3 of these angles, whereas the fourth can deviate by ± 45°^35^.

The β-turns, as defined by C.M. Venkatachalam, are characterized by a hydrogen bond between the N-H and C = O of residues *i* and *i*+3^36^. Venkatachalam has also defined types I, II, and III, and their corresponding mirror image types, I’, II’ and III’^36^. Crawford and collaborators have proposed a more strict definition in terms of distance^37^. Lewis and co-workers have added types V and V’. β-turn type VI is characterized by the presence of a proline; type VII is associated with a kink; and type IV corresponds to all other non-classified β-turns^38^. Different turns have been excluded for various reasons: β-turns III and III’ are too close to the 3_10_-helix and types I and I’, whereas turns V, V’ and VII are rare, and their definitions are inaccurate^35^. Type VI is divided into 2 sub-types, VI_a_ and VI_b_. Hutchinson and Thornton^39^ have divided type VI_a_ into the 2 sub-types VI_a1_ and VI_a2_. Wilmot and Thornton have precisely defined type VIII^40^, which is based on Richardson’s type I_b_ and was proposed after the removal of type VII^35^. The definitions used by Thornton’s group^39^,^41^ are currently considered to be the standard (see Supplementary Information 1)^42^. The β-turn assignment program PROMOTIF assigns β-turns on the basis of these standards^43^. Studies have shown that repetitive structure assignment approaches have a direct effect on decreasing or increasing the number of residues associated with β-turns^27^,^28^.

The difficulty with using such an approach is the ‘strict’ rule(s) used to define the β-turn types. Efimov has used a Ramachandran plot simplified to 6 and 8 regions: β (β_E_ and β_P_), γ, δ, α, ε and α_L_ (α_L_ and γ_L_). This rough clustering allows various classes to be defined, with some being associated with amino acid specific behaviours. The turns are also divided into full turns (with a polypeptide chain reversal of 180°) and half turns (with a polypeptide chain angle of 90°). The first category represents 7 major clusters, and the second one represents 8 major clusters^44^,^45^. This system has widely been used to define super-secondary elements^46^,^47^ and structural trees of protein superfamilies^48^,^49^,^50^. In a similar way, Wilmot and Thornton have also used a simplification of the Ramachandran plot for the following 6 major regions: β_E_, β_P_, α_R_, ε, α_L_ and γ_L_^51^. They observed 12 combinations in their dataset. The most frequent turns were easily detected, whereas the two most interesting non-classical turns were β_E_ → γ_L_ (8%) and γ_L_ → α_R_ (4%). The 6 other clusters represented only 1% each^51^.

More recently, Koch and Klebe have proposed a combination of turns of different lengths ranging from 3 to 6 residues; the turns sometimes overlap, thus leading to complex categorizations^52^. Koch and Klebe trained a very large modified Self-Organizing Map^53^,^54^ and extracted new types from the map. The assignment is provided as part of Secbase, an extension module of Relibase^55^. Koch and Klebe have used the identified new types in a second step to perform a prediction from the sequence^56^. This approach is innovative, but it has not been implemented as a web tool and is therefore less used. George Rose’s group has conducted research with a focus on the rationalization of two-, three-, and four-residue turn conformations found in their coil library^57^. Rose’s group has defined 12 categories and has used them in Monte-Carlo simulations. These categories cover at least 90% of coil library fragments ranging from 5- to 20-residues, thus indicating that longer fragments are composites of shorter ones^58^. Rose’s group has extended this approach to redraw the Ramachandran plot^59^.

However, none of these approaches has succeeded in superseding the classical definition of β-turns^35^,^36^,^41^,^43^. A major shortcoming of past β-turn classification concerns the classification of type IV β-turns, *i*.*e*., the miscellaneous category, because it represents 1/3^rd^ of β-turn residues and is the second most common type of β-turn. To locate potentially new recurrent conformations in this miscellaneous type, an automatic clustering approach based on the rules of β-turn assignment was designed. It is related to Self-Organizing Maps^53^,^54^ and takes into account the specificity of β-turn assignment rules. All type IV β-turns were clustered. The four most occurring clusters were chosen as new types and analysed. Unexpectedly, these sub-types, denoted IV_1_, IV_2_, IV_3_ and IV_4_, represent half of the type IV β-turns and occur more frequently than many of the classical types.

## Methods

### Data sets

To remove representative bias regarding protein resolution or sequence identity, non-redundant datasets were used. These datasets were generated using the PISCES database^60^. As previously performed in^12^,^61^, 10 sets of proteins were defined. Each contained no more than *x*% pairwise sequence identity (with *x* ranging from 20 to 90%). The selected chains had X-ray crystallographic resolutions less than 1.6 Å or 2.5 Å and R-factors less than 0.25 or 1.0. They comprised between 2,542 and 23,943 protein chains. Each chain was automatically examined with geometric criteria to avoid bias from zones with missing density. The main purpose of such diversity was to examine (i) the poorly populated turns and (ii) the stability of the clustering approach (see below).

### Secondary structure assignment

Secondary structure assignment was performed with DSSP^5^ (CMBI version 2000) using the default parameters. DSSP yields more than three states, so we reduced them to the following: the α-helix, containing α, 3_10_ and π-helices; the β-strand, containing only the β-sheet; and the coil, comprising everything else (β-bridge, hydrogen bond turn, bend, and coil). Turn assignment was performed as described previously^27^,^28^,^36^ using the following classical rules: the distance between residues *i* and *i*+3 should be less than 7 Å; the central residues of the turns must be non-helical; and in the case of strands, at least one residue must be associated with a coil. The types of turns (I, I’, II, II’, VI_a1_, VI_a2_, VI_b_ and VIII) were assigned according to the classical definition by using the φ and ψ dihedral angles of the central residues (see Supplementary Information 1). The turns were required to be less than 30° from the canonical values (at most one angle was allowed to deviate by +/− 45°)^43^. Types VI_a1_, VI_a2_ and VI_b_ were characterized by a cis-proline at position *i*+2. Turns that did not fit any of the above criteria were classified as type IV^39^,^43^. The turns were also classified into two classes according to their function as described by Efimov^44^,^45^: full turns resulting in a chain reversal of 180° and half turns that change the polypeptide chain direction by approximately 90°. This methodology was used to enable comparisons with previous studies.

### Protein Blocks

Protein Blocks (PBs^62^,^63^) corresponded to a set of 16 local prototypes, labelled from *a* to *p*, of 5 residue length that were described on the basis of dihedral angles (φ, ψ). The PBs were obtained with an unsupervised classifier similar to Kohonen Maps^54^ and hidden Markov models^64^. The PBs *m* and *d* are prototypes for the central regions of α-helix and β-strands respectively. PBs *a* through *c* primarily represent the N-cap of a β-strand, whereas *e* and *f* correspond to the C-caps; PBs *g* through *j* are specific to coils, PBs *k* and *l* correspond to the N cap of an α-helix, and PBs *n* through *p* correspond to C-caps. PBs were assigned by using in-house Python software, although similar assignment can be performed through the PBE web server^65^ or PBxplore (https://github.com/pierrepo/PBxplore^66^).

### Specific clustering approach

A specific clustering approach was designed to cluster type IV β-turns by using the classical rule, allowing +/− 30° for all angles, with the exception of one at +/− 45° for the defined values. The clustering derived from Self-Organizing Maps (SOM, without diffusion between the clusters^53^,^54^). The training was carried out in 2 successive parts; the first one limited the potential bias of initialization, and the second refined the clustering by using the specific rules for β-turn types. The type IV β-turns were selected from a dataset *D*. Thus, each dataset was associated with *T* type IV β-turns.

Step one:

1. *k* clusters were created and were vectors *v* of length 2*M* = 4, representing the dihedral angles (φ_i+1_, ψ_i+1_, φ_i+2_, and ψ_i+2_). *k* type IV β-turns were taken randomly to initialize the clusters.

2. One of the *T* type IV β-turns was randomly selected from the dataset *D* (denoted **V**_2_) and compared with each of the *k* clusters.

The dissimilarity measure between two vectors **V**_1_ (representing the clusters) and **V**_2_ of dihedral angles was defined as the Euclidean distance among the *M* links, the RMSDA (root mean square deviations on angular values^67^):


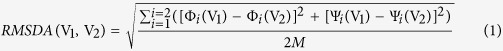


where {Φ_*i*_(**V**_1_), Ψ_*i*_(**V**_1_)}(resp. Ψ_*i*_(**V**_2_), Ψ_*i*_(**V**_2_)) denotes the series of the (2*M*) dihedral angles for **V**_1_ (resp. **V**_2_). The angle differences were computed modulo 360°. Thus, in the training, this distance was used for assessing the dissimilarity of any fragment in the database with the different clusters.

3. The minimal *RMSDA* value was used to define the winning cluster W, *i.e.*, the closest to the observation. W values were modified according to the learning coefficient α:


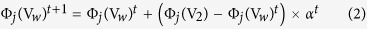



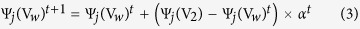


where {Φ_*j*_(V_*w*_)} and Ψ_*j*_(**V**_*w*_) are the values of the winner at time *t*, with *j* ranging from 1 to 2, similar to the values of the real data (*i.e.*, dihedral angles *i*+1 and *i*+2, modulo 360°).


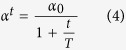


The decrease of α was performed similarly to that for SOM^53^,^54^, *T* represents the total amount of data to learn (here the number of type IV β-turns). *t* represents the number of β-turns already used. The process goes back to step 2. One cycle of training corresponds to the learning of the whole dataset α_0_, which is then equal to α_0_/2; after 5 cycles, it is equal to α_0_/5, etc. Initially, α_0_ = 0.35, as in^68^,^69^.

The process was iterated for 20 cycles, *i.e.*, 20 times *T*; these steps were important to diminish the potential effect of the initialization.

Step two:

1. The final values of the *k* clusters were used as initial values. α_0_ was still equal to 0.35.
2. One of the *T* type IV β-turns was randomly selected from the dataset *D* (denoted **V**_2_) and compared with each of the *k* clusters. Instead of using only RMSDA, the β-turn rule was used: 3 angles can be at +/− 30° and 1 angle at +/− 45°.The winner positively applied this rule; otherwise no training was performed.
3. Modification of the winner weights was performed as in step one −3.
4. The process was iterated for 20 cycles.

An important point is the choice of *k*. *k* was first set at 50 and then reduced. The obtained clusters were compared in the order of largest to smallest *k* values.

### Z-score

The amino acid occurrences for each local structure conformation were normalized into a Z-score:


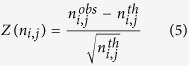


where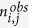 is the observed number of occurrences of amino acid *i* in position *j* for a given secondary structure, and 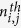 is the expected number. The product of the occurrences in position *j* with the frequency of amino acid *i* in the entire databank equals 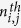. Positive Z-scores (respectively negative) corresponded to overrepresented amino acids (respectively underrepresented); threshold values of 4.42 and 1.96 were chosen (probability less than 10^−5^ and 5.10^−2^, respectively). The same computation was also performed for the protein blocks.

### Analysis

Most of the quantitative analysis was performed using in-house Python scripts, and statistics and visualization were performed with R software (version 3.2.2)^70^.

## Results and Discussion

### Protein structure dataset

**Figure 1:**
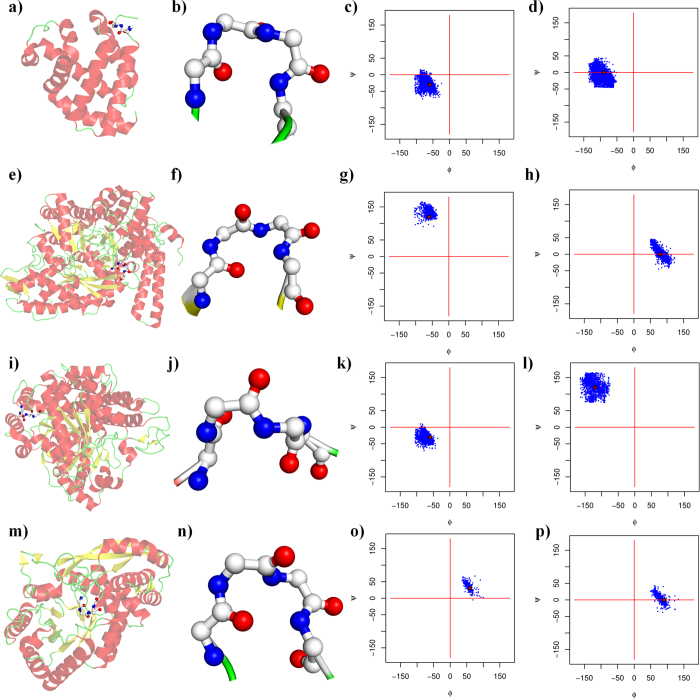
β-turn representation (beginning). (**a–d**) Type I, (**e–h**) type II, (**i–l**) type VIII, and (**m–p**) type I’ A turn close to the ideal values of its type (**a,e,i,m**) within a protein and (**b,f,j,n**) a close-up of the turn. Type I is represented by PDB id 2BK9^85^, type II by PDB id 1H16^86^, type VIII by PDB id 1SU8^87^ and type I’ by PDB id 1KKO^88^. (**c,g,k,o**) Ramachandran plot (φ, ψ) of residue *i*+1 and (**d,h,l,p**) of residue *i*+2; red dots are the ideal values. The number of observations of both residues is strictly identical.

**Figure 2:**
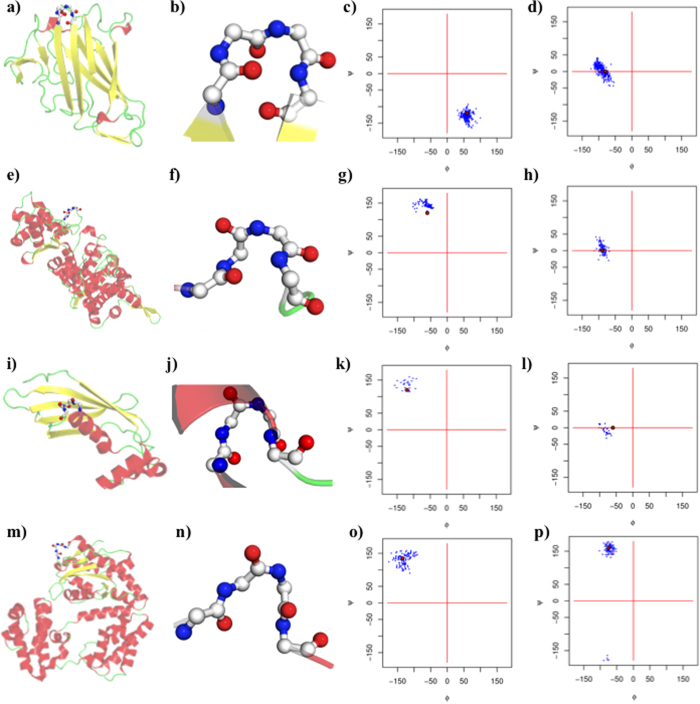
β-turn representation (end). (**a–d**) Type II’, (**e–h**) type VI_a1_, (**i–l**) type VI_a2_, and (**m–p**) type VI_b_ (see Fig. 1 for legend). Type II’ is represented by PDB id 1UXA^89^, type VI_a1_ by PDB id 1HBN^90^, type VI_a2_ by PDB id 1IQ6^91^, and type VI_b_ by PDB id 1YT3^92^.

The different amino acid datasets showed the expected amino acid and protein block occurrences, with no peculiarities in the rate of redundancy and the resolution quality (see Supplementary Information 2). As noted previously^27^,^28^, the occurrence of β-turns is highly dependent on the way in which the assignment is performed. Following the work of Fuchs and Alix^27^, we assigned secondary structures to the different protein datasets by using DSSP^5^. The DSSP provided 8 classes that were reduced to 3 classes (helix, strand and coil) or 4 classes (helix, strand, turn and coil, see Supplementary Information 3) for practicality. Helical structures represented more than 37.3% of the residues and the β-sheets represented 22.5%, whereas the remaining coil class covered 42.7% of the residues and included 20.4% of the β-turns (11.9% were turns and 8.5% were bends). Our β-turn assignment in the coil regions provided a slightly different number, with 21.9% being β-turns (difference: 1.5%). In total, 71.8% were similar to the DSSP assignment (45.6% were turns, and 23.0% were bends), whereas 28.1% and 1.9% were associated with coils and bridges, respectively. These proportions were comparable to the results of previous studies^27^,^28^. The β-turn types were then assigned by using classical definitions (described in the methods section, see Supplementary Information 1). Type I β-turns were the most frequent (38.2%), followed by the miscellaneous type IV (31.7%), and types II (11.8%), VIII (9.8%), I’ (4.1%), II’ (2.5%) and the different sub-types of the type VI β-turns (ranging from 0.9 to 0.2%, see Table 1). Henceforth, the type IV β-turns will be denoted type IV^ori^ to differentiate them from the new types in the current analyses. Figures 1 and 2 show the different types of β-turns in 3D and the distribution of their dihedral angles in the Ramachandran plot^36^,^71^,^72^.

### Analyses of discarded types

As a first step, before searching for new types, the previously discarded types were analysed.

Notably, type III and III’ β-turns had been included by Venkatachalam^36^, but have been discarded because they are considered to be too close to the 3_10_ helices and to type I (and I’) β-turns. The type V β-turn has been considered to be a rather unusual departure from the type II β-turn (see Figures 35 and 36 of ref. 35). If the type III β-turn were still recognized, it would represent 9.6% of the residues; *i.e.*, it would be the third most frequently occurring type. The obsolete type III’ β-turn represented approximately 1.5% of the turns, whereas the type V and V’ β-turns represented only 0.03 and 0.02%, respectively (see Supplementary Information 4), and were associated with type IV β-turns (see Supplementary Information 5), but they were negligible.

For the type III and III’ β-turns, the overlap with type I and I’ β-turns remained as expected, with 88.7% of the type III β-turns assigned as type I β-turns, 87.6% of the type III’ β-turns assigned as type I’ β-turns (see Supplementary Information 4 and 6), and the remaining 11–12% associated with type IV β-turns. Interestingly, 60% of type I β-turns were also assignable to type III, and 83.9% of type I’ were assignable to type III’ (see Supplementary Information 7). Therefore, the decision to remove this particular definition was clearly reasonable.

### Searching for new types

From the above section, it is apparent that nearly 1/3^rd^ of residues are not associated with a defined type. Moreover, as presented in the methods section, learning was performed on the type IV β-turns, the clustering was conducted on the basis of dihedral angles with an unsupervised approach similar to the approaches used for protein blocks^62^,^67^. The first step of learning was entirely unsupervised and was performed to properly define the initial values of the clusters, whereas the second step dictated the specific rules of the β-turns (*e*.*g*., +/−30° and one dihedral angle at +/− 45°).

A major difficulty in every classification approach is the choice of the clusters. Here, it was slightly different; the idea was not to have an optimal number of clusters but to assess the most frequently occurring and recurrent clusters to define the new pertinent types. In related research, Micheletti and collaborators have decided to take the largest cluster each time and iteratively repeat the clustering, each time removing the largest cluster^73^. This clustering is slightly unstable because each repetition removes a large amount of data. Thus, it did not seem pertinent to use it here. Moreover, with a large initial number of clusters, determining the *clusterability* of the data was manageable.

The training was performed with different datasets beginning with a large number of clusters (50 at first), which was progressively reduced (to 10). A notable feature of the learning was that four clusters appeared at the beginning and remained the most frequently occurring cluster for each of the different datasets. The deviation in the dihedral angle values between the different simulations (and different datasets) was never higher than 0.3°, thus indicating that the clustering was reasonably stable (a more detailed description is provided in Supplementary Information 8).

**Figure 3:**
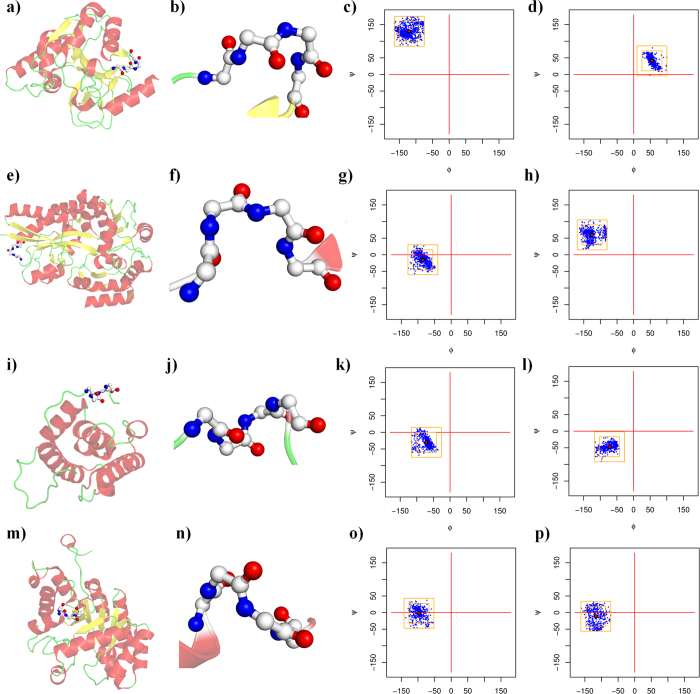
New β-turn representation. (**a–d**) Type IV_1_, (**e–h**) type IV_2_, (**i–l**) type IV_3_, and (**m–p**) type IV_4_ (see Fig. 1 for legend). Type IV_1_ is represented by PDB id 1JYK^93^, type IV_2_ by PDB id 1URS^94^, type IV_3_ by PDB id 1PA7^95^, and type IV_4_ by PDB id 1QWG^96^.

The four new type IV β-turn sub-types were named IV_1_, IV_2_, IV_3_ and IV_4_. They represent half of the of type IV β-turns (see Table 2), composing 16.1, 12.4, 11.2 and 8.5% of the IV^ori^ type, respectively. In regards to all of the defined types, they were the 4^th^, 6^th^, 7^th^ and 8^th^ most frequent turns (5.10%, 3.9%, 3.5% and 2.7%, respectively). These numbers are reasonable because they were highly consistent across all of the datasets. Figure 3 shows these four new categories. The remaining clusters were not selected because (i) their occurrences were very low (largely less than those of type VI β-turns) and (ii) they were often dependent on the number of clusters (see Supplementary Information 9). They were not useful for either protein structure or sequence–structure relationship analyses. The rest of the type IV β-turns were classified as IV_misc_.

**Figure 4:**
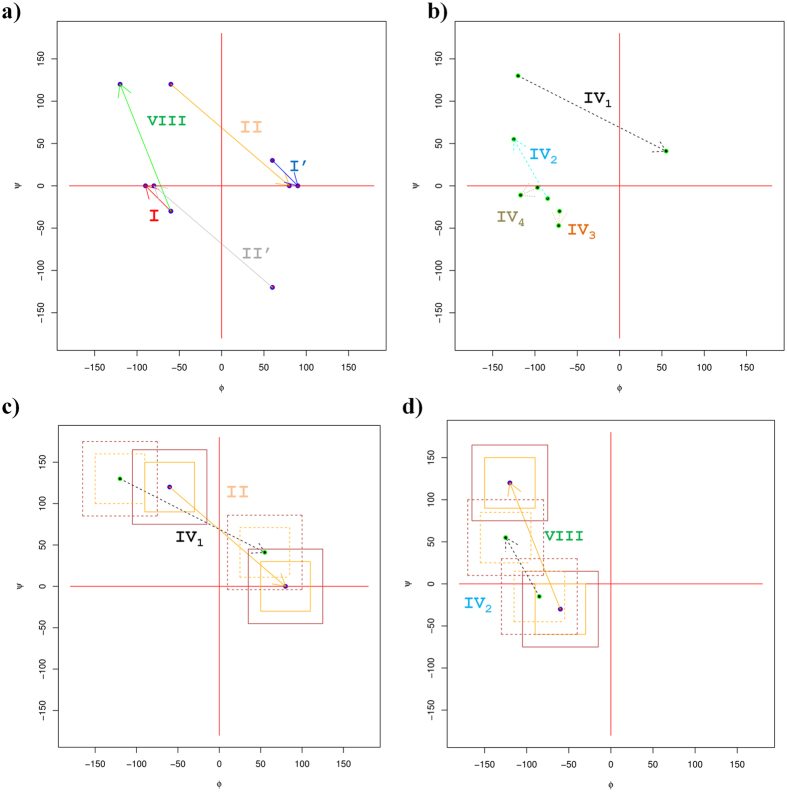
Ramachandran plot of the different β-turn types. An arrow connects the dihedral angle values of residue *i*+1 to residue *i*+2. (**a**) Classical β-turns, (**b**) new β -turns, (**c**) a close-up of type II and IV_1_ β -turns, and (**d**) on type VIII and IV_2_ β -turns, the first square corresponds to the +/− 30° rule, and the second one to the +/− 45° rule.

Table 3 provides the observed angles. Because the clustering approach was based on the specific clustering of type IV, no overlap could be found with the existing types. Figures 4a,b show the relative position of each turn. A relationship was observed between type IV_1_ and type II β-turns (see Fig. 4c) and between type IV_2_ and VIII β-turns (see Fig. 4d, see Supplementary Information 10). In terms of dihedral angle values, the type IV_1_ β-turn resembled a slightly displaced conformation of the type II β-turn, whereas the type IV_2_ β-turn appeared to be a less extended type VIII β-turn. Type IV_3_ and IV_4_ were much more specific, with very particular dihedral angles in the helical regions (see Supplementary Information 11).

### New turns in regards to DSSP

To describe the type IV β-turns more precisely, we examined their former DSSP assignments (hydrogen bond estimation) as turns or bends. Interestingly, more than 2/3 of the residues of IV^ori^ were identified by DSSP as turns, with 35% being bends and 37% being hydrogen bond turns, and the rest were mainly associated with coils and β-sheets. The type IV_misc_ was more associated with non-hydrogen bond, stabilized local structures, with a 41% enrichment in bends and 31% fewer hydrogen bond turns. This evolution is mainly associated with the newer and less frequent type IV β-turns (*e.g.*, type IV_3_ and IV_4_), which comprise 70% and 49% hydrogen bond turns. The evolution was strikingly lower for the type IV_1_ β-turn, with less than 30% of residues associated with hydrogen bond turns. Although all the new type IV β-turns were linked to neither α-helices nor β-sheets, type IV_1_ β-turns were often observed at the ends of β-sheets (in nearly 2/3 of the cases).

### Comparison with previous analyses

As mentioned in the introduction, two major efforts were made in the 1980 s and 1990 s to define β-turns. Both were based on a Ramachandran plot divided into 6 to 8 large regions. The size and shape of these regions were largely different from the strict rule of +/−30° (and 45°). Notably, these previous classifications were performed with all turns, whereas in the current analyses the classification was performed on only a subset of type IV β-turns.

Table 4 shows the new turns classified using a Ramachandran plot division scheme similar to that described above. Efimov has proposed a very precise definition of turns and half-turns with 7 and 8 types of turn^44^,^45^. Interestingly, type IV_1_ might seem as if it could be characterized as β_E_α_L_ because it looks like the proposed βαL-half-turn; however, the type IV_1_ β-turn is not a half-turn but a complete turn. The type IV_3_ β-turn is the only local conformation that can be described as a half-turn, but instead of being a αγ-half-turn, it is mainly α/γ- > α. Type IV_4_ β-turns can be described as γγ; a similar type has been described in^45^, but here it is mainly a turn, whereas the previously described types were half-turns. In fact, the type IV_2_ β-turns were the only ones that seemed to be directly related to Efimov’s analyses, because they could be characterized by a γδ connection between α-helices, as described in^45^. The percentage of turns and half-turns observed correctly correlated with the distance threshold proposed by Crawford and co-workers^37^.

Wilmot and Thornton have also used a simplification of the Ramachandran plot in 6 major regions, with 12 combinations^51^. Because the size of the different regions is higher than Efimov’s, the number of types is relatively limited. The region α_R_ represents the γ, δ and α regions; very diverse conformations were found in type IV_3_ and IV_4_ β-turns as well as type I β-turns (*i.e.*, α_R_ → α_R_). Type IV_2_ β-turns had the same description as type VIII (*i.e.*, α_R_ → β_E_). Interestingly, only two non-classical turns, β_E_ → γ_L_ (8%) and γ_L_ → α_R_ (4%)^51^, were defined by Wilmot and Thornton. One could expect that one of these two types might be associated with the most frequent new turn. However, this was not the case, because the type IV_1_ β-turn is not β_E_ → γ_L,_ but β_E_ → α_L_.

Hence, these comparisons illustrate that the specific clustering performed in the current analyses highlighted one new main cluster that was not observed previously: the type IV_1_ β-turn. Additionally, it showed the specificity of the type IV_3_ and IV_4_ β-turns in regards to their fine description. The type IV_2_ β-turn was the only one to have been clearly characterized previously by both studies^45^,^51^.

Koch and Klebe (KK) used a sophisticated approach to unify the assignment of turns of different lengths^52^. This approach is not easily comparable to others because: (i) it is not based on the classical assignment rules and (ii) all the turns have been re-assigned. Hence, for β-turns, other features were used in the training in addition to the values of the dihedral angles (φ, ψ) of the central residue. Classical and new β-turns were compared to the final definition of the 24 open KK β -turns (7 were considered to be *non-turn-like structures*) and 18 reverse KK β–turns presented in Supplemental Data S14 and S16 of ref. 52. Owing to the particular learning method, type I’, II and II’ β-turns had no direct equivalent in the KK β-turns, whereas type I, IV_3_ and IV_4_ β-turns were associated with the KK type I β-turn (18% of the true turns). Type VIII β-turns were associated with the KK type VIII3 β-turn (6.5% of the true turns). Interestingly, type IV_2_ β-turns were not associated with any KK β-turn types.

Hence, this comparison between studies indicated some similarities because the major turn (type I β-turn) could not distinguish between the two new less frequent turns (types IV_3_ and IV_4_ β-turn), whereas type VIII β-turns were easily found by using this approach. Similarly to previous results, the type IV_2_ β-turn remained specific to our clustering. However, differences between the studies should be taken into account, such as the different learning method used by Koch and Klebe, considered more angles than ours and their training was conducted on the complete set of turns and not just the type IV β-turns.

### Comparison with protein blocks

Table 5 shows the over- and under-representation of protein blocks for all the β-turn types. Type IV^ori^ β-turns were characterized by a PB motif of [*efghijko*] [*bhijklno*] [*abghijlnop*] [*acgiop*]. As expected, this signature was more ambiguous in regards to the well-defined types, which showed a range of only one to four PBs at each position. The IV_misc_ represented only half of the previous β-turn IV^ori^ types. The only exception was the newly over-represented PBs *n* and *p* at positions *i* and *i*+1 as well as the reduced over-representation of PBs *n* and *p* at positions *i*+1 and *i*+3, whereas 28/32 over-representations remained the same.

The newly defined type IV β-turns had stronger PB motifs. They could be analysed not only in regards to β-turn IV^ori^ but also in regards to II and VIII for types IV_1_ and IV_2_.

For type IV_1_, the PB motif is [*aegp*] [*aegho*] [*hikp*] [*ail*] and has no direct contradiction with the classical behaviours of β-turn IV^ori^. However, this motif had some interesting specificities in regards to type IV_2_. However, the PB motifs of type II β-turns were less ambiguous, with only two main PBs at each position [*eg*] [*ho*] [*ik*] [*al*]. Type IV_1_ β-turns were clearly different, with 8 over-represented PBs that were under-represented in type II β-turns (PBs *a* and *p* at position *i*, PBs *a*, *e* and *g* at position *i*+1, PBs *h* and *p* at position *i*+2 and PBs *i* at position *i*+3). Similarly, in type IV_2_ β-turns, the PB motif was [*fjkl*] [*bklno*] [*bglp*] [*cg*] and was comparable to the type IV^ori^ β-turns but also had some differences compared with the type VIII β-turns. Hence, only half of the over-represented PBs in type VIII β-turn were found in type IV_2_ β-turns and 5 under-represented PBs were over-represented (PBs *k*, *n* and *p* at position *i*+1, and PBs *b* and *p* at position *i*+2).

PB motifs of type IV_3_ and IV_4_ β-turns were mainly associated with the most frequent β-turn, the type I β-turn, because their dihedral angles were in the same restricted area.

### Amino Acid Specificities of the new types

β-turns have been widely analysed in terms of sequence – structure relationships, which have been incorporated in various prediction approaches^27^,^74^,^75^. Table 6 shows the under- and over-represented amino acids in each type of turn. Some associations were expected because all of the different type VI β-turns were characterized by the proline at position *i*+2.

**Figure 5:**
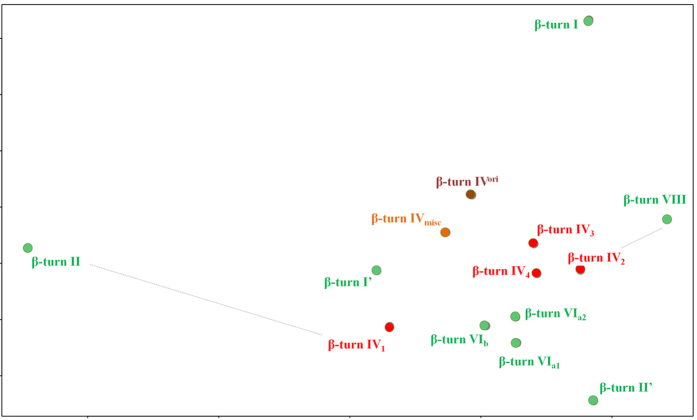
Sammon map of amino acid behaviours of the different β-turns. Classical turns are in green while new turns are in red.

Concerning the new turns defined in the current analyses, the four important points are as follows:

1. Type IV^ori^ and IV_misc_ β-turns remained strongly linked, because erasing half of the occurrences did not change the general trend of the unassigned turns.
2. IV_3_ and IV_4_ were clearly distinct in terms of dihedral angle distributions but had very similar amino acid compositions. Indeed, they shared the same over- or underrepresented amino acid trends in 80% of the cases; only one inversion of amino acid preference was observed for the type IV_3_ β-turns at position *i*+2 (alanine),
3. The type VIII and IV_2_ β-turns were structurally close, with high sequence similarity. We found only one inversion between these types at position *i*+2 for the valine residue.
4. Interestingly, the type IV_1_ and II β-turns were close structurally but had strongly divergent sequences. At position *i*, no common amino acid over- or under-representation was observed. In the Ramachandran plot’s α_L_ region, glycine represented 88% of the residues, whereas in γ_L_, it was only 38% (with N 17%, D 9%, K 5%, E and R 4%, respectively). Interestingly, the type IV_1_ encompassed mainly the non-glycine residues at *i*+2 (see Table 4). Moreover, proline and glycine residues were under-represented at position *i*+3 of type II, although they were over-represented in type VIII β-turns. Additionally, the *i*+2 positions of both types had more divergent residues. Figure 5 shows a Sammon map projection^76^ of all the β-turns. It emphasizes these relationships and highlights the strong differences between types IV_1_ and II, with the distance being quite substantial. The type IV_1_ β-turn amino acid composition was similar to that of the two other new β-turn types, IV_3_ and IV_4_ (see Supplementary Information 12 and 13).

## Conclusions

β-turns are the most important secondary structures preceded by the α-helix and β-sheet. β-turns correspond to approximately 25 to 30% of all protein residues^77^. The current classification of the different β-turns has remained unchanged for the past 30 years. In the 1980 s and 1990 s, different studies proposed extending the definition of turns, mainly on the basis of the division of a Ramachandran plot into 6 to 8 regions^46^,^51^,^78^. These analyses of β-turns showed strong similarities with classical analyses and provided new definitions for the least frequently occurring turns. Two recent studies have expressed interest in redefining the definitions: (i) Koch and Klebe^52^ have used a very large modified Self-Organizing Map^53^,^54^ and (ii) George Rose’s group has defined 12 categories comprising different lengths^57^,^58^. Nonetheless, these approaches were performed in a manner comparable to the secondary structure assignment that is still dominated by DSSP^5^. Although different turn classifications have subsequently been proposed^9^, none of them have been successfully used. The main idea in this study was not to redraw a novel classification but to extend the classical classification.

From an unsupervised classification, based exclusively on dihedral angles, four new types were defined. The two most frequently occurring, type IV_1_ and IV_2_ β-turns, were similar to existing type II and VIII β-turns but had very distinct features. On the one hand, type IV_2_ and VIII β-turns shared striking amino acid compositional features, with minor differences. However, type IV_2_ β-turns were associated with stabilizing hydrogen bonds, unlike type VIII β-turns. On the other hand, type IV_1_ and II β-turns were very close in terms of dihedral angles but were distinct in terms of their amino acid content. Figure 5 clearly shows that type II β-turns were highly specific, whereas type IV_1_ β-turns had more classical characteristics, being closer to type I’ β-turns than type II β-turns.

The two remaining β-turn types, IV_3_ and IV_4_, were within bin 6 of the Ramachandran plot, close to type I β-turns^79^. Although their amino acid profiles were highly similar, their local protein structure conformations were distinct.

A classical question raised by any clustering methodology is the relevance of the results. Here, our results can be considered reliable, owing to their reproducibility and stability. The use of 10 different datasets ranging in quality and sequence identity highlighted the high stability of the four main clusters (*i.e.*, the new turns). For each simulation, the clusters were always found at similar frequencies and with similar dihedral values. However, the other clusters were substantially more variable. A simple analysis was also performed to evaluate the possibility of the presence of sub-clusters inside the different clusters by diminishing the authorized dihedral angle deviation allowed during the training. Similarly, the centre of the four main clusters always appeared, thus supporting their stability.

Comparisons with the previous alternative classification proposed by Efimov^45^,^78^ and Thornton’s group^51^ emphasized the uniqueness of the approach. Notably, the most frequent new turn (type IV_1_ β-turn) was not highlighted, although it is the 5^th^ most occurring turn (including type IV_misc_ β-turns). Only the type IV_2_ β-turns were previously included.

This extended classification is relevant because it does not modify the currently accepted β-turn types, is highly stable (in regards to amino acid redundancy and the quality of protein resolution), and proposes new ways to analyse the architecture and dynamics of the protein or peptide structure of β-turns. Hence, we envision two potential applications of this classification system. The first one addresses molecular dynamics simulations in which researchers follow the dynamic evolution of type VIII β-turns^80^. The change from type VIII to a type IV (*i.e*., IV^ori^) during the simulations is very different when the turn is in fact a type IV_2_ or IV_misc_. The former case (type IV_2_ β-turn) is a simple extension of this conformation, whereas the latter (type IV_misc_ β-turn) is really a different independent conformation^80^. The second example involves an analysis of conformational characteristics of asparaginyl residues in proteins^81^. Interestingly, many are associated with turn conformations. With this new classification, only 16.5% (see Supplementary Information 14) were associated with miscellaneous turns (e.g., IV_misc_); thus, this classification provides a better description of local protein conformations and resolves the spectrum of IV_misc_ turns to a greater extent.

An interesting point is that turns are often observed as tandem repeats, sometimes leading to long series of γβ, βγ, ββ or γγ turns^82^. It is also notable that γ and β turns are associated with the same residues^83^,^84^. In future work, we plan to investigate the succession of turns, particularly the ones mentioned in this study.

## Additional Information

**How to cite this article**: de Brevern, A.G. Extension of the classical classification of β-turns. *Sci. Rep.* **6**, 33191; doi: 10.1038/srep33191 (2016).

## Supplementary Material

Supplementary Information

## Figures and Tables

**Table 1:** β-turn frequencies.

β-turn	(%)
I	38.21
II	11.81
VIII	9.84
I'	4.10
II'	2.51
VI_b_	0.88
VI_a1_	0.73
VI_a2_	0.20
IV^ori^	31.72
Sum	100.00

Classical types and their frequencies. Type VI_a1_, VI_a2_ and VI_b_ β-turns are characterized by a cis-proline at position *i*+2. Type IV is denoted IV^ori^ to distinguish it from the new classification.

**Table 2:** β-turn frequencies.

new β-turn	(%)	(%) of β-turn IV^ori^
IV_1_	5.10	16.08
IV_2_	3.95	12.44
IV_3_	3.53	11.15
IV_4_	2.70	8.50
IV_misc_	16.44	51.83
Sum	31.72	100.00

The four new β-turns are denoted IV_1_ to IV_4_, and the remaining residues are assigned to type IV_misc_. Their frequencies in regards to the turns and to the original type IV^ori^ are provided.

**Table 3:** New β-turns.

β-turn	*φ*_*i+1*_	*ψ*_*i+1*_	*φ*_*i+2*_	*ψ*_*i+2*_
Type IV_1_	−120.0	130.0	55.0	41.0
Type IV_2_	−85.0	−15.0	−125.0	55.0
Type IV_3_	−71.0	−30.0	−72.0	−47.0
Type IV_4_	−97.0	−2.0	−117.0	−11.0

Dihedral angle values of the four new turns.

**Table 4:** Torsion angle regions taken from Wilmot and Thornton, and Efimov, with turns and half-turn proportions as defined by Efimov and distance in regards to Crawford.

	Thornton (1990)		Efimov (1986)		Efimov (1986)		Crawford (1973)
	*i*+1	*i*+2	*i*+1	*i*+2	turns	half-turns	d < 5.7 Å
β-turn IV_1_	β_E_	α_L_	β_E_	α_L_	99.4	0.6	23
β-turn IV_2_	α_R_	β_E_	ϒ	δ	72	28	37
β-turn IV_3_	α_R_	α_R_	ϒ/α	α	71	29	54
β-turn IV_4_	α_R_	α_R_	ϒ	ϒ	65	35	28
*β-turn IV*^*misc*^					64	36	19

**Table 5:** Protein blocks’ Z-scores of β-turn types.

		*i*	*i*+1	*i*+2	*i*+3
β-turn I	(+)	FJKL	KLN	BGLOP	CGOP
	(−)	aBCDEGHIMNOP	ABCDEFGHIJMOP	ACDEFHIJKM	ABDEFHIjKL
β-turn II	(+)	EG	HO	IK	AL
	(−)	aBCDFHjKLMnOP	ABCDEFgIjKLMNP	ABCDEFghjLMnO	BCDEFghiKMNoP
β-turn VIII	(+)	AFKP	ABL	CDGL	bCFK
	(−)	BCDEgHMNO	CDEFgHIjKMNop	aBefhIjKMnoP	ADIjLMnoP
β-turn I'	(+)	EGHjNO	HO	IP	A
	(−)	bcDfklMp	abcDefklM	bcDefhkMo	bcDefhklMp
β-turn II'	(+)	abHO	J	ABLO	CGLP
	(−)	DfM	abcDfkMo	cDfkmp	adkm
β-turn VI_a1_	(+)	Cdp	EF	BHK	BIL
	(−)	fkM	dkM	cdflM	ckM
β-turn VI_a2_	(+)	Bi	aeFg	BK	gjlo
	(−)	m	m	m	m
β-turn VI_b_	(+)	bCdj	aCD	Df	bDf
	(−)	fkM	fklM	klM	clM
β-turn IV^ori^	(+)	EFGHiJKO	BHIJKLNO	ABGhIjLNOP	ACGiOP
	(−)	BCDM	CDFM	CDeFkM	DEfklM
β-turn IV_1_	(+)	AEGp	aEGHo	HIKP	AIL
	(−)	cDfhklMo	bcDfklMnp	abcDflMo	cDkMnp
β-turn IV_2_	(+)	FJKL	BKLno	bGLP	Cg
	(−)	bcDehmop	CDFghiMp	acDeFhiKm	aDeiL
β-turn IV_3_	(+)	FjK	KL	BLmNo	cGMnOp
	(−)	bCDehinop	abCDeFhiop	aCDeFhik	abDeFhikl
β-turn IV_4_	(+)	FJK	KLN	BGLOP	CGoP
	(−)	bDehmno	acDfhiMp	acDefhikM	abDefhiklm
β-turn IV_misc_	(+)	EFGHIjkNO	BHIJLNOP	ABGIJLnOP	AbCgjP
	(−)	bCDM	CDeFM	cDfkM	DeM

**Table 6:** Amino acid’s Z-score of β-turn types.

		*i*	*i*+1	*i*+2	*i*+3
β-turn I	(+)	cPghStND	PSEK	whSTNDe	wcGn
	(−)	IVLmAfywQERK	IVLmFywcqGhtn	IVLmAPG	ivlmqPErk
β-turn II	(+)	qP	Pek	GN	mcqSt
	(−)	sD	ivLywcGst	IVLmAFywqPSTdERK	ilPgn
β-turn VIII	(+)	acPGs	PDek	IVFyhNd	ivP
	(−)	Ivlmqerk	ilfycGh	lAPG	lafGe
β-turn I'	(+)	Fst	GNd	Gn	yqr
	(−)	Q	ivpt	ivlapsterk	p
β-turn II'	(+)	St	G	stN	mg
	(−)	Vlafqpter	P	ivlp	
β-turn VI_a1_	(+)	Vp	afYp	P	fyg
	(−)		ilt		ip
β-turn VI_a2_	(+)	N	ne	P	h
	(−)				
β-turn VI_b_	(+)	P	Y	P	pr
	(−)	G	ivlagstderk		
β-turn IV^ori^	(+)	CPGStnD	PGsndk	gHtND	PGTn
	(−)	IVlaqerk	IVLmAc	IVLaqP	ivLAdek
β-turn IV_1_	(+)	cG	hnek	GhND	cpG
	(−)	Ve	***g***	ivlap*t*k	d
β-turn IV_2_	(+)	PgS	pstDk	hND	P
	(−)	Ivlmrk	ivafg	ivlP***G***	vladek
β-turn IV_3_	(+)	CsnD	***a***P	vmt	fystn
	(−)	Ivak	cq	p***g***	iqe
β-turn IV_4_	(+)	Cpgsnd	fptnD	whtNd	fGh
	(−)	Vle	iva***g***	lp***g***	***p***ek
β-turn IV_misc_	(+)	cPgnd	PGn	GhtNd	pgTn
	(−)	Ivlar	iVLmAfc	ivLay	vla

Colors underline the difference of new turns and the original type IV^ori^ (underline new over- or under representation, in bold italics inversion of over- or under representation, see Methods section).
