# Supplementary material for: Extension of the classical classification of β-turns
Source: Sci Rep. 2016 Sep 15;6:33191. doi: 10.1038/srep33191 (PMC5024104; doi:10.1038/srep33191)
Supplement: Supplementary Information [file srep33191-s1.doc]

**Supplementary Information**

**Extension of the classical classification of β-turns**

### Alexandre G. de Brevern^1,2,3,4,*^

^1^ INSERM, U 1134, DSIMB, F-75739 Paris, France.

^2^ Univ Paris Diderot, Sorbonne Paris Cité, UMR_S 1134, F-75739 Paris, France.

^3^ Institut National de la Transfusion Sanguine (INTS), F-75739 Paris, France.

^4^ Laboratoire d'Excellence GR-Ex, F-75739 Paris, France.

| β-turn | φ_i+1_ | ψ_i+1_ | | φ_i+2_ | | ψ_i+2_ |  |
| --- | --- | --- | --- | --- | --- | --- | --- |
| I | -60.00 | -30.00 | | -90.00 | | 0.00 |  |
| I’ | 60.00 | 30.00 | | 90.00 | | 0.00 |  |
| II | -60.00 | 120.00 | | 80.00 | | 0.00 |  |
| II’ | 60.00 | -120.00 | | -80.00 | | 0.00 |  |
| IV ^a^ | ---- | ---- | | ---- | | ---- |  |
| VI_a1_ ^b^ | -60.00 | 120.00 | | -90.00 | | 0.00 |  |
| VI_a2_ ^b^ | -120.00 | -120.00 | | -60.00 | | 0.00 |  |
| VI_b_ ^b^ | -135.00 | | 135.00 | -75.00 | 160.00 | | |
| VIII | -60.00 | -30.00 | | -120.00 | | 120.00 |  |

**Supplementary Information 1.** *Values of dihedral angles of β –turns*^1-4^.

^a^ Turns which do not fit any of the above criteria are classified as type IV.

^b^ Types VI_a1_, VI_a2_ and VI_b_ are characterized by a cis-proline (*i*+2).

1 Hutchinson, E. G. & Thornton, J. M. PROMOTIF--a program to identify and analyze structural motifs in proteins. *Protein Sci* **5**, 212-220 (1996).

2 Chan, A. W., Hutchinson, E. G., Harris, D. & Thornton, J. M. Identification, classification, and analysis of beta-bulges in proteins. *Protein Sci* **2**, 1574-1590 (1993).

3 Venkatachalam, C. M. Stereochemical criteria for polypeptides and proteins. V. Conformation of a system of three linked peptide units. *Biopolymers* **6**, 1425-1436 (1968).

4 Richardson, J. S. The anatomy and taxonomy of protein structure. *Adv Protein Chem* **34**, 167-339 (1981).

1. **(b)**

| amino acids | (%) |
| --- | --- |
| I | 5.50 |
| V | 7.04 |
| L | 8.87 |
| M | 2.04 |
| A | 8.71 |
| F | 4.09 |
| Y | 3.54 |
| W | 1.52 |
| C | 1.35 |
| Q | 3.81 |
| P | 4.68 |
| G | 7.75 |
| H | 2.38 |
| S | 5.76 |
| T | 5.60 |
| N | 4.33 |
| D | 5.93 |
| E | 6.52 |
| R | 4.97 |
| K | 5.59 |
| **Sum** | **100.00** |

| Protein Blocks | (%) |
| --- | --- |
| *a* | 3.86 |
| *b* | 4.09 |
| *c* | 7.79 |
| *d* | 17.95 |
| *e* | 2.32 |
| *f* | 6.41 |
| *g* | 1.09 |
| *h* | 2.26 |
| *i* | 1.76 |
| *j* | 0.78 |
| *k* | 5.31 |
| *l* | 5.30 |
| *m* | 31.01 |
| *n* | 2.15 |
| *o* | 2.82 |
| *p* | 3.48 |
| **Sum** | **100.00** |

**Supplementary Information 2.** *Dataset properties*. (a) Amino acid and (b) Protein Blocks distribution.

(**a) (b)**

| three-state secondary structures (%) | |  | eight-state secondary structures (%) | |
| --- | --- | --- | --- | --- |
| α-helix | 37.37 |  | α-helix | 33.29 |
|  |  |  | 3_10_ helix | 4.05 |
|  |  |  | π-helix | 0.03 |
| coil | 42.72 |  | coil | 19.13 |
|  |  |  | β-bridge | 1.17 |
| *(turns* | *20.37)* |  | turn | 11.84 |
|  |  |  | bend | 8.53 |
| β-sheet | 21.53 |  | β-sheet | 21.53 |
| **Sum** | **100.00** |  | **Sum** | **100.00** |

**Supplementary Information 3.** *Secondary structure frequencies*. (a) with the three-state definition (with the turns inside the brackets), and (b) with the eight-state definition.

| (%) | turn I | turn I' | turn IV | Sum |
| --- | --- | --- | --- | --- |
| turn III | 8.54 | 0.00 | 1.09 | 9.63 |
| turn III' | 0.00 | 1.28 | 0.18 | 1.46 |
| turn V | 0.00 | 0.00 | 0.03 | 0.03 |
| turn V' | 0.00 | 0.00 | 0.02 | 0.02 |
| Sum | 8.54 | 1.28 | 1.32 | 11.14 |

**Supplementary Information 4.** *Occurrence of discarded β-turns*. Is provided the percentage of turns now assigned to β-turn types I, I’ and IV. Only 11% of (ex-) β-turn types III is not assigned to β-turn types I and only 12% of (ex-)β-turn types III’ not to β-turn types I’.


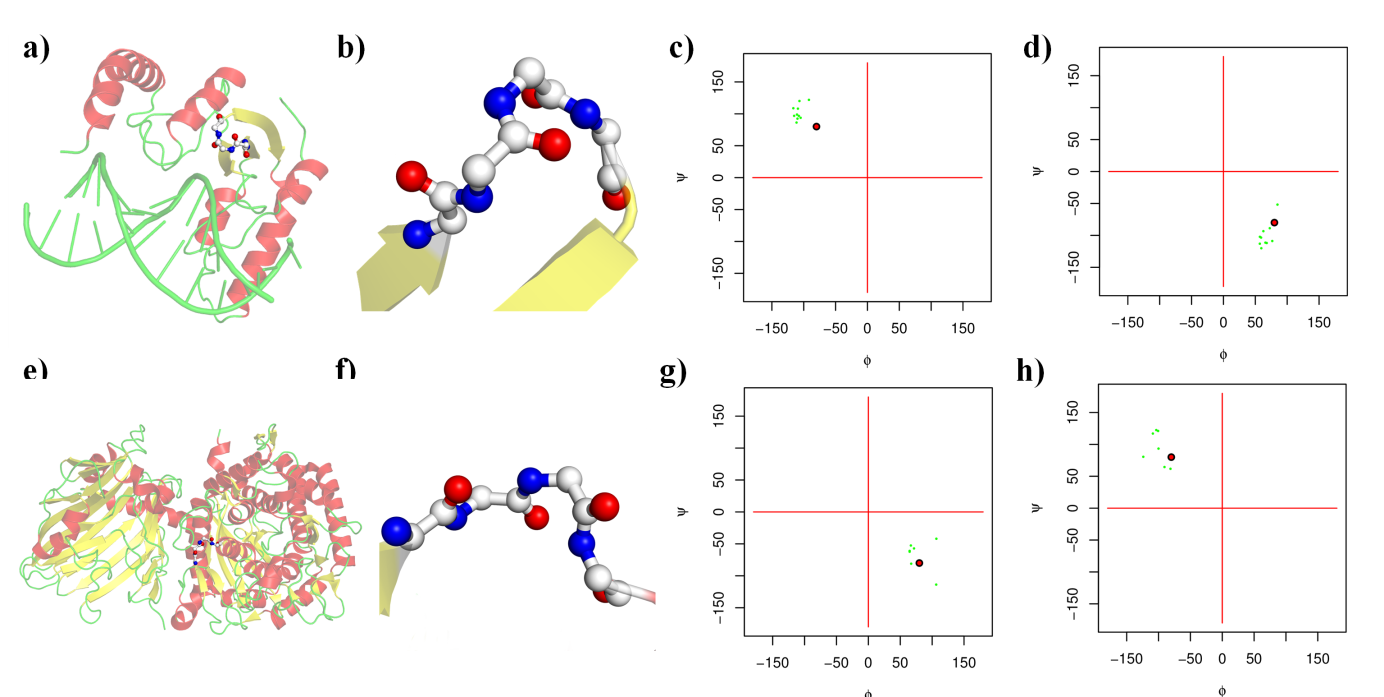


**Supplementary Information 5.** *The (ex-)β-turn types V and V’*. (a-d) Type V and (e-h) type V’. A turn close to the ideal values of its type (a, e) within a protein (PDB id [3PVI](http://www.rcsb.org/pdb/explore.do?structureId=3pvi) ^1^ and [1V7W](http://www.rcsb.org/pdb/explore/explore.do?structureId=1V7W) ^2^, resp.) and (b, f) a close-up of the turn. Ramachandran plot (c, g) of residue *i*+1 and (d, h) of residue *i*+2. They are all associated to type IV. Red dots are the ideal values.

1 Horton, J. R., Nastri, H. G., Riggs, P. D. & Cheng, X. Asp34 of PvuII endonuclease is directly involved in DNA minor groove recognition and indirectly involved in catalysis. *J Mol Biol* **284**, 1491-1504, doi:10.1006/jmbi.1998.2269 (1998).

2 Hidaka, M. *et al.* Chitobiose phosphorylase from Vibrio proteolyticus, a member of glycosyl transferase family 36, has a clan GH-L-like (alpha/alpha)(6) barrel fold. *Structure* **12**, 937-947, doi:10.1016/j.str.2004.03.027 (2004).


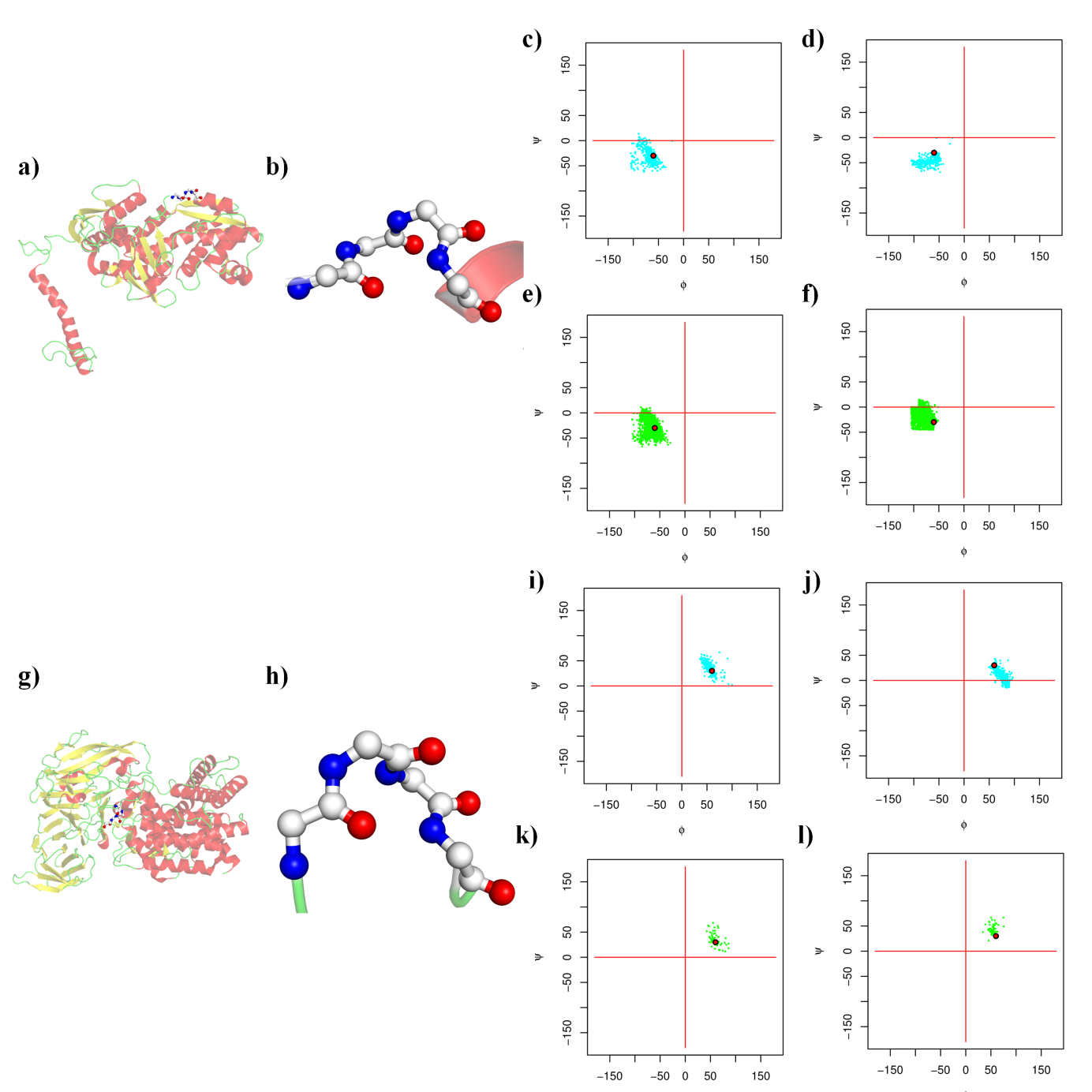


**Supplementary Information 6.** *The (ex-)β-turn types III and III’*. (a-f) Type III and (g-h) type III’. A turn close to the ideal values of its type (a, g) within a protein (PDB is [1K92](http://www.rcsb.org/pdb/explore/explore.do?structureId=1K92) ^1^ and [1RWH](http://www.rcsb.org/pdb/explore/explore.do?structureId=1RWH) ^2^, resp.) and (b, h) a close-up of the turn. Ramachandran plot (c, e, i, k) of residue *i*+1 and (d, f, j, l) of residue *i*+2. In light blue, these β-turns are associated (c, d) to type I and (i, j) to type I’, while in green, (e, f, k, l), they are assigned to type IV.

1 Lemke, C. T. & Howell, P. L. The 1.6 A crystal structure of E. coli argininosuccinate synthetase suggests a conformational change during catalysis. *Structure* **9**, 1153-1164 (2001).

2 Lunin, V. V. *et al.* High-resolution crystal structure of Arthrobacter aurescens chondroitin AC lyase: an enzyme-substrate complex defines the catalytic mechanism. *J Mol Biol* **337**, 367-386, doi:10.1016/j.jmb.2003.12.071 (2004).


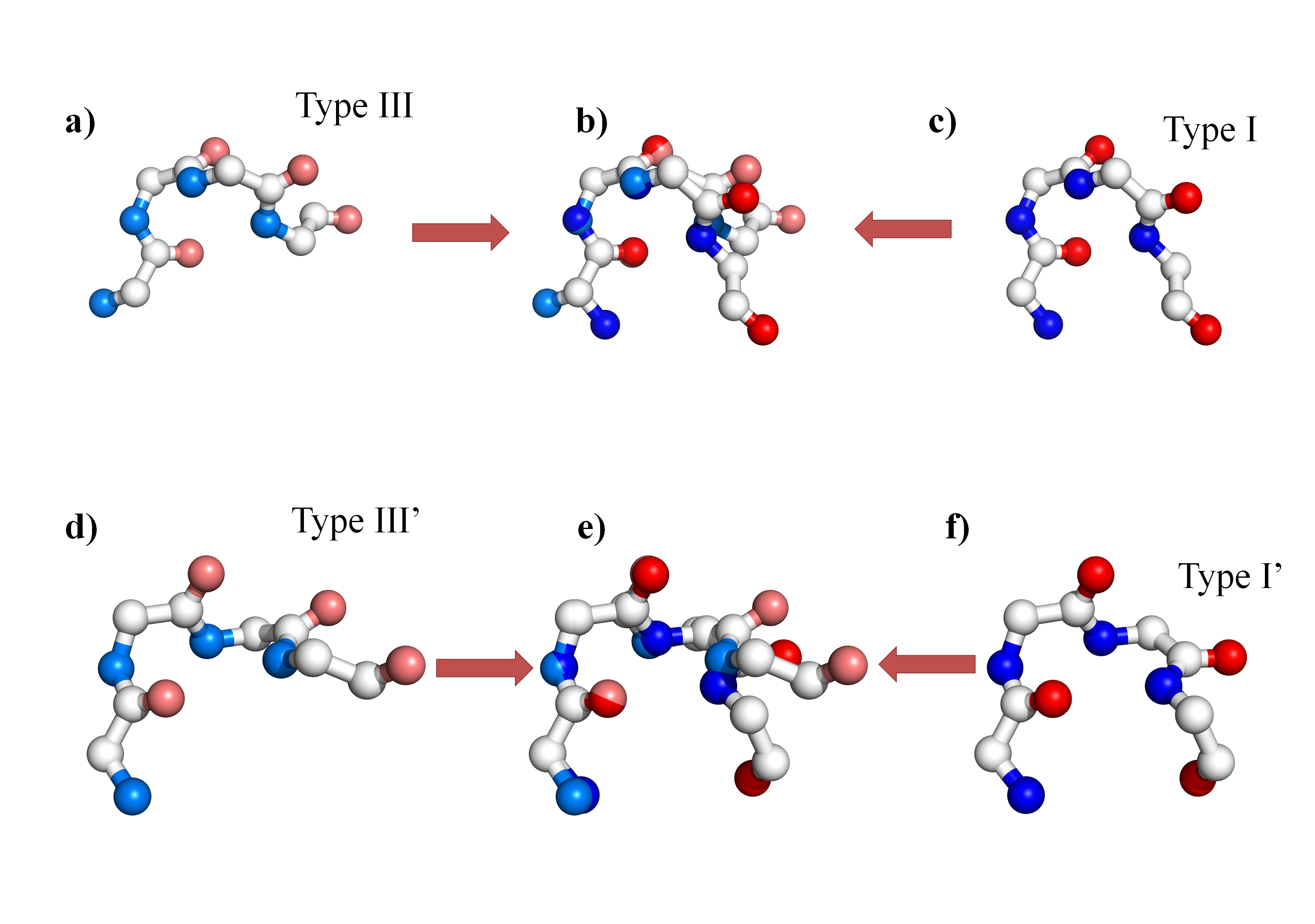


**Supplementary Information 7.** *Superimposition of discarded β-turns*. (a) (ex-) β-turn type III is (b) superimposed to (c) β-turn type I, while (d) (ex-) β-turn type III’ is (e) superimposed to (f) β-turn type I’.

**Supplementary Information 8.** *Details on the clustering process*.

Following an efficient approach previously used^1^, the number of clusters was chosen high (50). With a large number of clusters, their sizes were small and also highly unstable. Comparison of the centres (the dihedral angle values) between different simulations shows a large variation. Moreover, some clusters were also overlapping due to the rules used (+/- 30° and one with 45°).

The number of clusters was reduced from 50 to 5. The choice of 10 was dictated by (i) one cluster appeared with an occurrence higher than 10% and (ii) no overlapping was observed. 100 independent simulations had been performed with random initial values. The simulation with the minimum distance with the all the other simulations was taken as the initial values for the centers of the clusters. 100 new simulations were performed.

Obtained clusters were compared. The four main clusters appeared each times with a very high stability (>99% of the turns were always associated to these same four most important clusters), see ^2^ for more details. The following less representative clusters showed a high volatility, they were not stable. Moreover, they were associated to low occurrence.

These 4 clusters were used for each datasets to test their stability. It remains equivalent for every kind of resolution and sequence identity.

1. de Brevern, A. G., Etchebest, C. & Hazout, S. Bayesian probabilistic approach for predicting backbone structures in terms of protein blocks. Proteins 41, 271-287 (2000).
2. Celton M., Malpertuy A., Lelandais G., de Brevern A.G. Comparative analysis of missing value imputation methods to improve clustering and interpretation of microarray experiments. BMC Genomics11(1):15 (2010).

**
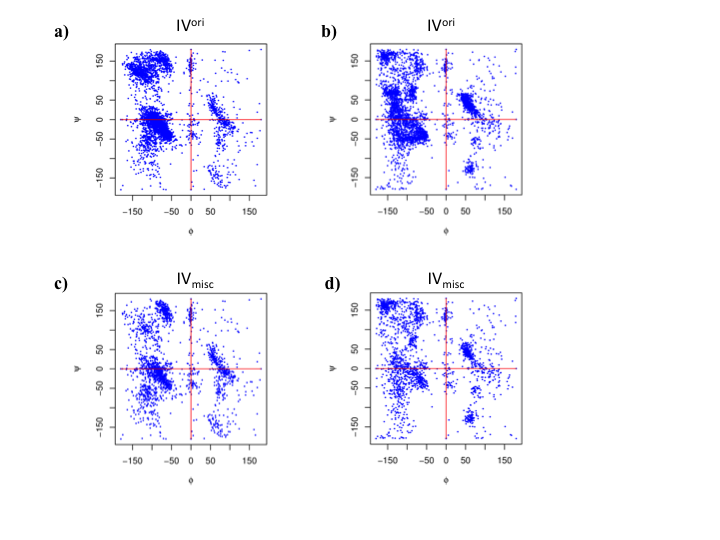
**

**Supplementary Information 9.** *Ramachandran plots.* (a-b) β turn IV^ori^ and (c-d) β turn IV_misc_. (a, c) for residue *i*+1 and (b, d) for residue *i*+2.


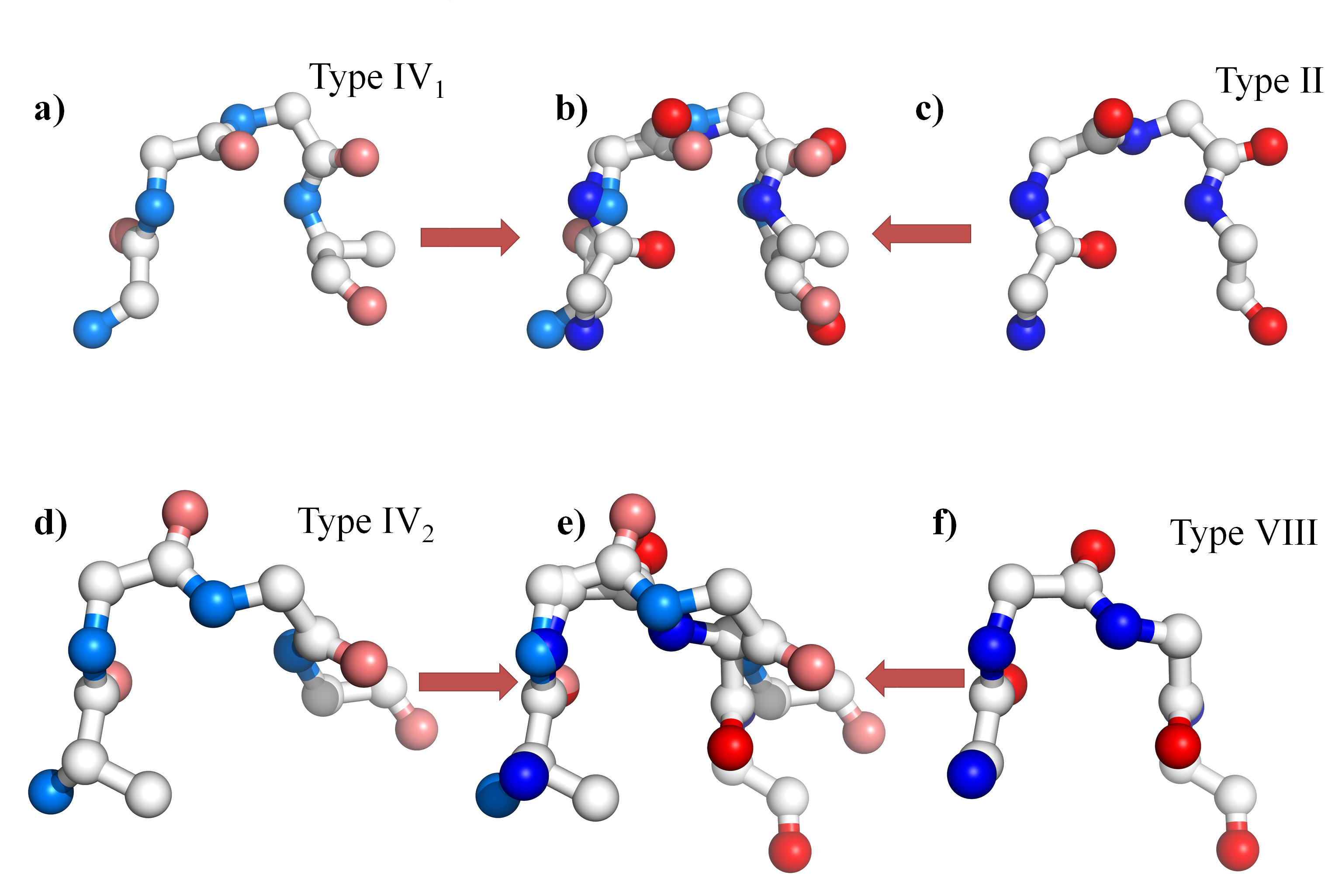


**Supplementary Information 10.** *Superimposition of new β-turns with most related turns*. (a) β-turn type IV_1_ is (b) superimposed to (c) β-turn type II, while (d) β-turn type IV_2_ is (e) superimposed to (f) β-turn type VIII.

As expected, β-turn type IV_1_ is superimposable to β-turn type II, differences being a little bit on every part of the β-turn type. β-turn type IV_2_ has local similarity with β-turn type VIII, but on a global level, it is more different. It is also due to the Ramachandran regions encountered by this turn that is more extended.


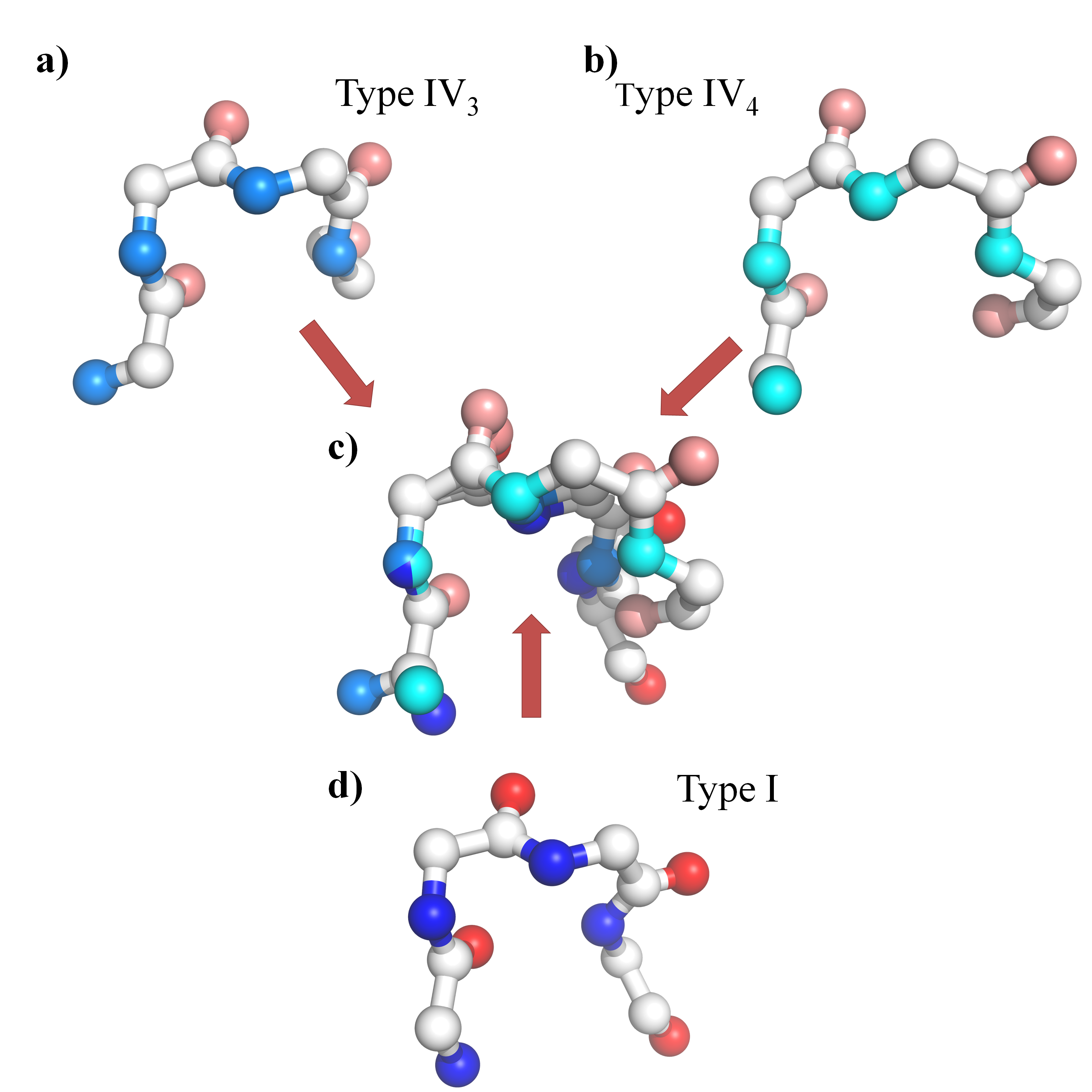


**Supplementary Information 11.** *Superimposition of new β-turns with most related turns*. (a) β-turn type IV_3_ and type IV_4_ are (c) superimposed to (f) β-turn type I.

This representation underlines the specificities of each turn. While they share some similarities for the central residues, their extremities have specific behaviours making them different.


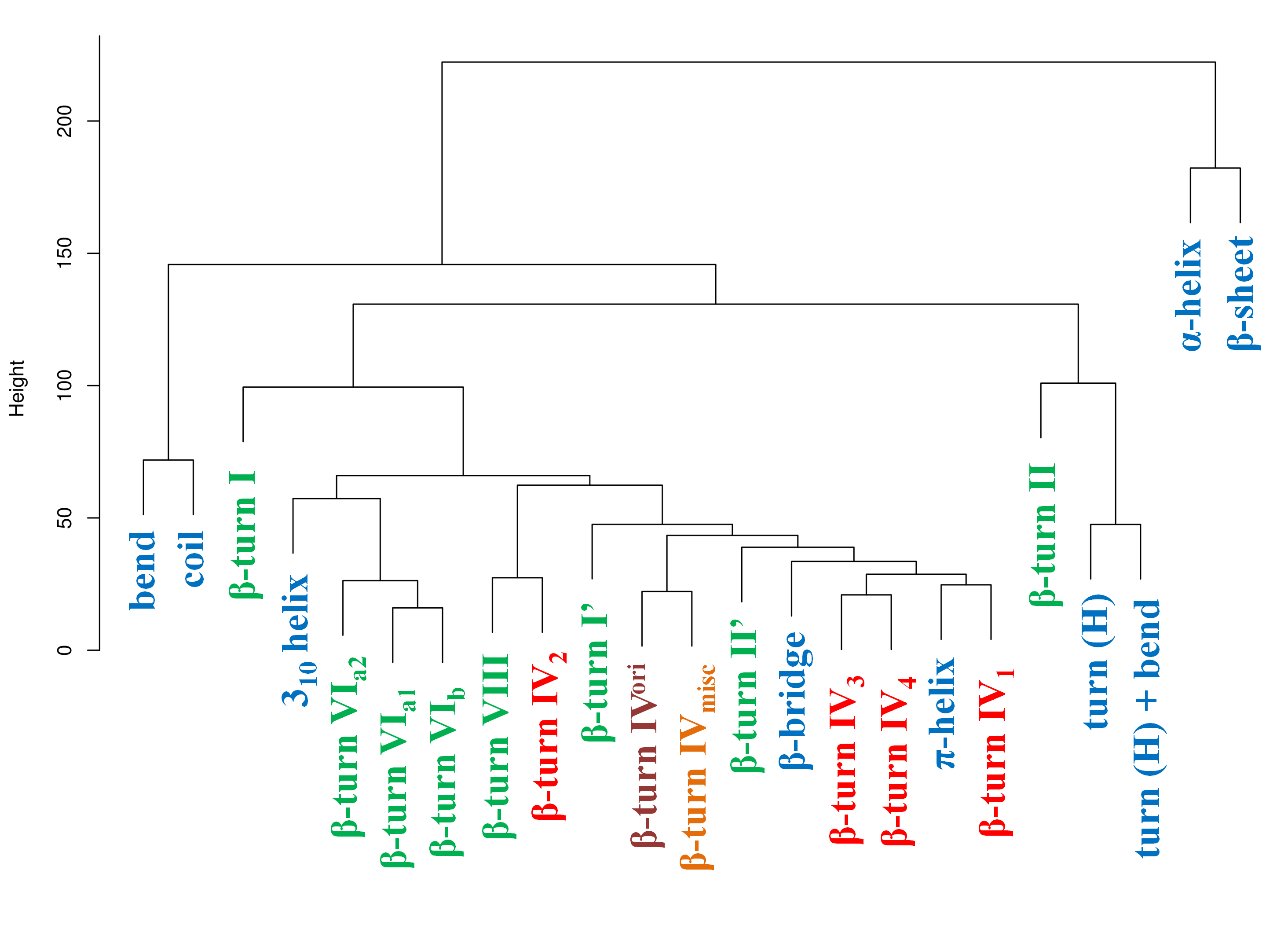


**Supplementary Information 12.** *Relation of amino acid propensities between the different kinds of secondary structures*. A hierarchical clustering of the amino acid propensities had been performed on the eight-state secondary structure assigned by DSSP (blue colour). The turns (as defined in Method section) had been classified with classical types (in green) and new types (in red), while original turn IV (β-turns IV^ori^) and new one (β-turns IV_misc_) are respectively in brown and orange.


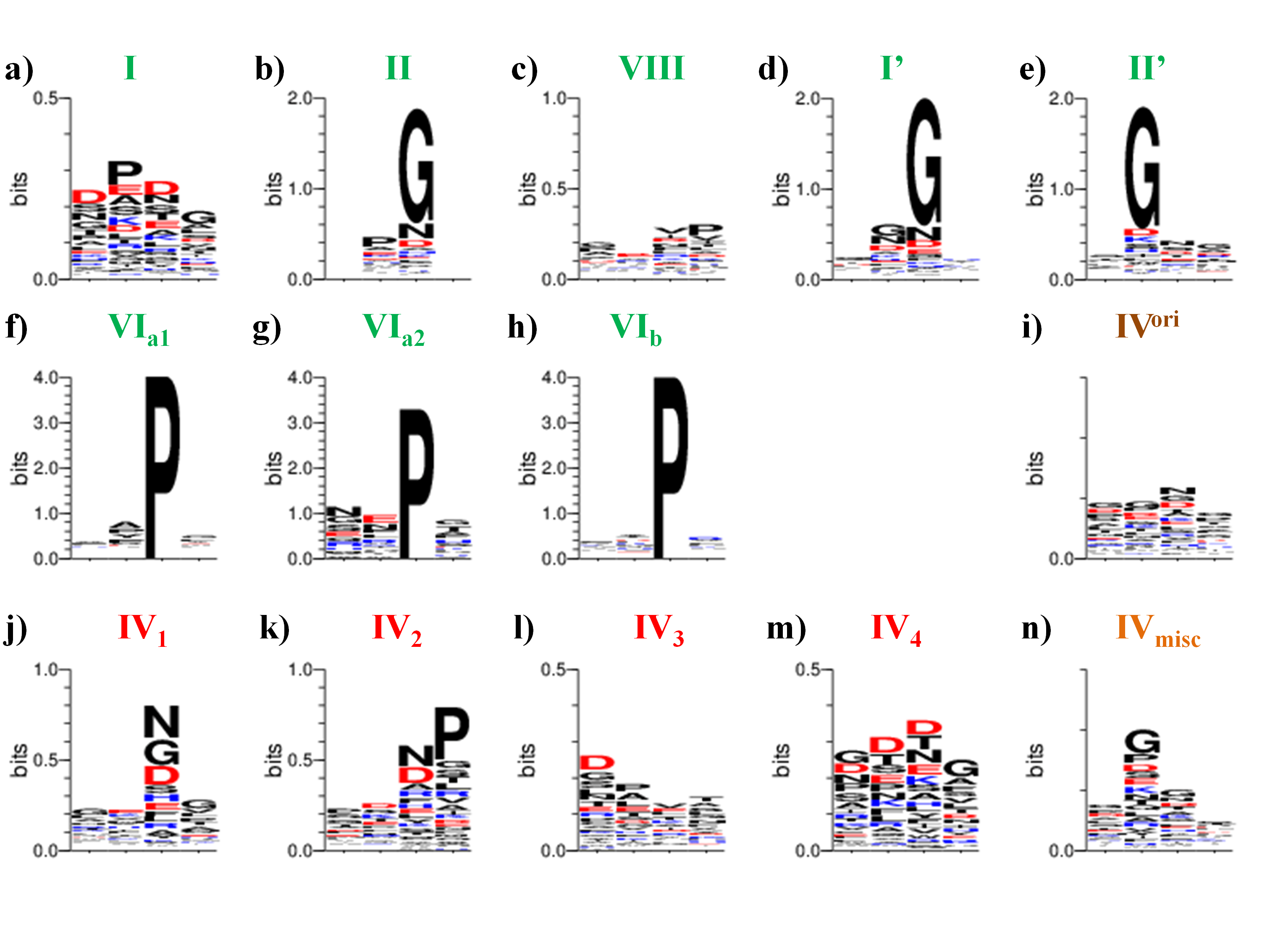


**Supplementary Information 13.** *Amino acid propensities between the types of turns*. (a-f) classical types, (i) original type IV (β-turns IV^ori^), (j-m) the four new types and (n) remaining type IV (β-turns IV_misc_). The amino acid frequencies are shown using [WebLogo 3.4](http://weblogo.threeplusone.com/manual.html) ^1^.

1 Crooks, G. E., Hon, G., Chandonia, J. M. & Brenner, S. E. WebLogo: a sequence logo generator*. Genome R*e**s** 14, 1188-1190 (2004).

|  | β-turn type | (%) |
| --- | --- | --- |
| 1 | I | 38.21 |
| *2* | *IV^ori^* | *31.72* |
| *2* | *IV_misc_* | *16.44* |
| 3 | II | 11.81 |
| 4 | VIII | 9.84 |
| 5 | IV_1_ | 5.10 |
| 6 | I' | 4.10 |
| 7 | IV_2_ | 3.95 |
| 8 | IV_3_ | 3.53 |
| 9 | IV_4_ | 2.70 |
| 10 | II' | 2.51 |
| 11 | VI_b_ | 0.88 |
| 12 | VI_a1_ | 0.73 |
| 13 | VI_a2_ | 0.20 |

**Supplementary Information 14.** *Occurrence of β-turns including the new classes*. The classic β-turns are coloured in green and the new ones are in red. The original turn IV (β-turns IV^ori^) and new one (β-turns IV_misc_) are respectively in brown and orange.
